# Effect of High Voltage Cold Plasma on Oxidation, Physiochemical, and Gelling Properties of Myofibrillar Protein Isolate from Asian Sea Bass (*Lates calcarifer*)

**DOI:** 10.3390/foods10020326

**Published:** 2021-02-04

**Authors:** Oladipupo Odunayo Olatunde, Avtar Singh, Khursheed Ahmad Shiekh, Pornpot Nuthong, Soottawat Benjakul

**Affiliations:** 1 International Center of Excellence in Seafood Science and Innovation, Faculty of Agro-Industry, Prince of Songkla University, Hat Yai, Songkhla 90110, Thailand; oladipupo.olatunde177@gmail.com (O.O.O.); avtar.s@psu.ac.th (A.S.); skhursheed2015@gmail.com (K.A.S.); 2 Office of Scientific Instrument and Testing, Prince of Songkla University, Hat Yai, Songkhla 90110, Thailand; pornpot.n@psu.ac.th

**Keywords:** protein oxidation, non-thermal technology, myofibrillar protein isolate, gel, cold plasma, fish

## Abstract

The effects of in-bag dielectric barrier discharge high voltage cold plasma (IB-DBD-HVCP) on myofibrillar protein isolate (MPI) from Asian sea bass (ASB) and its impact on the physiochemical and gelling properties of MPI gels were elucidated. A mixture of argon (90%) and oxygen (10%) was used for generating IB-DBD-HVCP. MPI was subjected to IB-DBD-HVCP for varying times (5–15 min). Total carbonyl content was increased, while total sulfhydryl content was decreased in MPI, especially with augmenting treatment time (TT) (*p* < 0.05). Surface hydrophobicity initially increased when IB-DBD-HVCP TT of 5 min (DBD-HVCP5) was implemented, followed by subsequent decrease with increasing TT. Based on gel electrophoresis, lower actin and myosin heavy chain (MHC) band intensities were found for MPI subjected to IB-DBD-HVCP, particularly when a TT longer than 10 min was used, compared to those of the control. Gel made from DBD-HVCP5 had higher breaking force, deformation, and highest G′ value compared to others. A more ordered and fibrous network was found in DBD-HVCP5 treated gel. Therefore, IB-DBD-HVCP treatment, particularly for 5 min, enhanced cross-linking of proteins in ASB myofibrillar proteins, which resulted in the improved gel elasticity and strength.

## 1. Introduction

Thermal or non-thermal technologies have been used for processed seafood, mainly for preservation or shelf-life extension [1]. During thermal treatment, protein denaturation and protein oxidation are induced, causing alterations in seafood, particularly in the techno-functional properties as well as the acceptability of seafood or seafood products [2]. The application of non-thermal technologies (NTTs) to preserve food quality, particularly during processing as well as storage, is gaining more attention. NTTs including pulsed light, high-intensity ultrasound, ionizing radiation, oscillating magnetic fields, ultraviolet light, high hydrostatic pressure, and pulsed electric fields as well as high voltage cold plasma (HVCP) have been introduced for seafood [3]. These technologies have shown promising potential in inactivating spoilage and pathogenic microorganisms in seafood without significant changes in organoleptic properties, while maintaining the food nutritive value [4,5]. Nonetheless, some negative changes toward quality, particularly protein alteration, occur depending on the type and condition of the non-thermal technology used.

The excitement of any gas (combined or single) with electric field or energy exceeding the gas ionization potential will change its state to the ionized form, known as plasma. During this process, diverse species such as negative and positive ions, radicals, neutral and excited molecules, electrons, and quanta of electromagnetic radiation (i.e., ultraviolet and visible light) are generated [6,7]. HVCP technology has rapidly evolved as a novel non-thermal preservation technology for seafood including fish [8] and shrimps [9,10]. HVCP generates several reactive species (RESPE) including reactive nitrogen species (RNS) as well as reactive oxygen species (ROS) such as peroxide, nitrogen oxides (NxOy), singlet oxygen, and ozone, which are responsible for their excellent bacteria inhibition [11]. Nevertheless, RESPE have been documented to induce the oxidation of lipid and protein in seafood [12,13]. 

Protein isolates, which are the most refined form of concentrated protein, are very digestible and easily incorporated into different food products [14]. Protein isolates from seafood have shown promising potential in the production of new formulated foods [15]. Nevertheless, the effect of non-thermal technology, particularly HVCP, have not been elucidated. Protein oxidation (PT-OX), which is the covalent modification of protein mediated by RESPE produced by HVCP, could play a direct (interaction of the protein with RESPE) or indirect (interaction of the protein via the secondary oxidation products) role in protein deterioration [16]. The backbone of protein and the side chain of amino acids undergo oxidation, when exposed to RESPE [17]. These could induce protein–protein cross-linkages and/or protein fragmentation [16]. Single backbone cleavage mediated by the oxidation of the side chains of aspartyl, prolyl, and glutamyl could induce protein fragmentation [18]. The modifications of sulfur containing amino acids (methionine and cysteine) also initiated PT-OX and these modifications can change the protein properties (techno-functional) including structure, enzyme activities, susceptibility to proteolysis, and solubility [16]. Additionally, PT-OX can increase the risk of some diseases, affect their digestibility as well as decrease nutritional value [19]. Similarly, modifications of protein, either by crosslinking or fragmentation induced by PT-OX could influence the meat quality including texture, solubility, and water holding capacity (WHC) [16]. Recently, most published research has focused on the antimicrobial efficacy of HVCP in seafood with the aim of shelf-life extension. There is limited information on the effect of PT-OX induced by HVCP on the quality of seafood protein, especially the functional properties. Therefore, the impact of oxidation induced by HVCP on the properties of Asian sea bass (ASB) myofibrillar protein isolate (MPI) was elucidated in this study. Furthermore, the effect of PT-OX induced by HVCP on the resulting gel properties of ASB-MPI was also investigated. 

## 2. Materials and Methods

### 2.1. Chemicals

Analytical grade chemicals used in the study were purchased from Sigma (St. Louis, MO, USA), while the chemicals used for electrophoresis were procured from Fluka (Buchs, Switzerland).

### 2.2. Procurement and Preparation of Myofibrillar Protein Isolate (MPI)

Fresh Asian sea bass (ASB) were brought from Songkhla Lake’s fish farm and were transported to the laboratory in crushed ice (1:3, *w*/*w*). Immediately after arrival, ASB were washed with cold distilled water (CDW), drained, and minced. The prepared mince was added with CDW (3:1, *w*/*w*), the resulting mixture was gently stirred for 10 min in a low storage room (4 °C), and the washed mince was filtered with a layer of nylon screen. This process was repeated three times. Thereafter, MPI was prepared from the washed fish mince by the alkaline solubilization method detailed by Kobayashi et al. [20], in which washed mince was homogenized (1 min at 8000 rpm) with CDW (1:9, *w*/*v*). Using 2 N NaOH, the pH of the mixture was adjusted to 11. Samples were then centrifuged at 4 °C for 20 min at 8000× *g*. To the supernatant, the pH was adjusted to the isoelectric point (pH 5.5) using 2 N HCl. The precipitate was collected, and the pH was adjusted to 7 using 2 N NaOH and named as ‘myofibrillar protein isolate (MPI)’. A hundred grams (100 g) of the MPI was then placed on a polystyrene foam tray and spread to obtain a uniform layer. The prepared samples were then inserted into 18 × 28 cm^2^ low-density polyethylene bags laminated with polyamide with a thickness of 0.070 mm. The mixture of argon (90%) and oxygen (10%) using a paste/gas ratio of 1:3 (*v*/*w*) was filled in the bags with the aid of a Henkovac type 1000 (Tecnovac, Grassobbio (BG), Italy) and heat sealed. The oxygen concentration in the sealed bag was monitored using the OxyBaby headspace analyzer (WITT Gasetechnik, Witten, Germany). 

### 2.3. The Impact of High Voltage Cold Plasma (HVCP) on Protein Oxidation (PT-OX) of MPI

The HVCP system consisted of a high voltage transformer (input voltage 230 V at a frequency of 50 Hz) and voltage variac (output voltage controlled within 0~260 V). HVCP discharge was generated between two 15-cm diameter aluminum electrodes separated by two Perspex dielectric layers (15 mm thickness). The system was operated at high voltage (80 kVRMS) under atmospheric pressure. InfiniVision 2000 X-Series Oscilloscope (Agilent Technologies Inc., Santa Clara, CA, USA) was used to monitor the input current and voltage characteristics of the system. Between the two dielectric layers, a low-density polypropylene bag was placed, which served as both an additional dielectric barrier and sample holder. A constant distance of 2.0 cm was kept between the electrodes for all trials. In-bag dielectric barrier discharge HVCP (IB-DBD-HVCP) was used for sample treatment [21,22]. The sealed bags containing MPI were positioned between the ground cathode and the quartz. IB-DBD-HVCP treatment was conducted at room temperature (28 ± 2 °C) for different times (5, 10, and 15 min). MPI with the temperature of 4 °C was subjected to IB-DBD-HVCP treatment. The temperature of MPI treated for 15 min was still below 10 °C. Immediately after treatment, bags containing the MPI were kept at 4 °C for 1 h to allow the contact between reactive species and MPI. The sample without IB-DBD-HVCP was used as the control (CON). All samples were subjected to analyses.

Total sulfhydryl content was determined using 5,5′-dithiobis(2-nitrobenzoic acid) (DTNB) [23]. Surface hydrophobicity was determined using 1-anilinonaphthalene-8-sulphonic acid (ANS) as a probe [24]. 

Protein patterns of samples were analyzed under a reducing condition (in the presence of BME) using sodium dodecyl sulphate-polyacrylamide gel electrophoresis (SDS-PAGE) [25]. First, samples (3 g) were mixed with 27 mL of 5% SDS solution. The mixture was homogenized at a speed of 5000 rpm for 2 min and the homogenate was incubated at 85 °C for 30 min. The mixture was centrifuged for 20 min at 12,000× *g*. The supernatant (15 µg protein) was subjected to SDS-PAGE analysis. Quantitative analysis of the protein band intensity was performed using a Model GS-700 Imaging Densitometer (Bio-Rad Laboratories, Hercules, CA, USA) with Molecular Analyst Software version 1.4 (image analysis systems).

Total carbonyl content (TCC) was determined as tailored by Chanarat et al. [26]. 

Fourier transform infrared (FTIR) spectra were also determined [27]. FTIR spectra of MPI were obtained using a Bruker Model Invenio S FTIR spectrometer (Bruker Co., Ettlingen, Germany). About 10 mg of dried MPI samples were mixed with 90 mg potassium bromide (KBr), creating a pellet. The absorption of IR in the region of 400–4000 cm^−1^ was determined using 32 scans with a resolution of 4 cm^−1^. Background (pure KBr) was subtracted using the Opus software (Bruker Instruments, Billerica, MA, USA). Additionally, these spectra were subtracted from the reference spectrum of air. The peaks were identified by software and assigned according to the literature values.

### 2.4. The Impact of In-Bag Dielectric Barrier Discharge High Voltage Cold Plasma (IB-DBD-HVCP) on the Gelling Properties of MPI

#### 2.4.1. Gel Preparation

Gels from MPI without and with IB-DBD-HVCP treatment for different times were prepared following the procedure of Singh et al. [28]. The MPI were mixed with 2.5% NaCl and blended for 2 min. Thereafter, the final moisture content of the MPI paste was adjusted to 80% using CDW and blended for 2 min. Then, the paste was filled into a polyvinyl casing and both ends were tightly sealed. After setting for 30 min at 40 °C, heating was done for 20 min at 90 °C. The resulting gels were cooled down in CDW for 1 h and left in the refrigerator overnight before analyses.

#### 2.4.2. Analyses

Textural, Physical, and Rheological Properties

Breaking force (BF) and deformation (DF) were determined using a texture analyzer (Stable Micro Systems, Surrey, UK) using a spherical probe of 5 mm in diameter [29]. Whiteness and expressible moisture content were also assayed [30]. Dynamic rheology of MPI containing 2.5% NaCl using a rheometer (HAAKE RheoStress1, ThermoFisher Scientific, Karlsruhe, Germany) was determined [31].

#### 2.4.3. Microstructure

Microstructures of gel prepared from MPI without and with IB-DBD-HVCP treatment for various times were examined using a scanning electron microscope (SEM) (Quanta 400; FEI, Eindhoven, the Netherlands). The method of Quan and Benjakul [30] was used for sample preparation prior to visualization. The images were recorded. 

### 2.5. Statistical Analysis

Completely randomized design (CRD) was used throughout the study. Data obtained from replicated experiments (*n* = 3) were subjected to one-way analysis of variance (ANOVA) and means were compared by Duncan’s Multiple Range Test (DMRT). SPSS package (SPSS 20.0 for Windows, SPSS Inc, Chicago, IL, USA) was used for data analysis. 

## 3. Results and Discussion 

### 3.1. Effect of IB-DBD-HVCP on Asian Sea Bass (ASB) MPI

#### 3.1.1. Total Carbonyl Content (TCC), Surface Hydrophobicity (SHP), and Total Sulfhydryl Group Content (TSHC)

The effect of IB-DBD-HVCP treatment for different times on total carbonyl content (TCC), surface hydrophobicity (SHP), total sulfhydryl group content (TSHC) of the MPI is presented in Table 1. TCC was increased with augmenting IB-DBD-HVCP treatment time (TT), in which the values increased from 1.16 to 7.93 nmol/g protein (*p* < 0.05). These were in tandem with the finding of Olatunde et al. [32], who documented an increased TCC for ASB muscle protein when subjected to IB-DBD-HVCP. Carbonyls, which are derived from the oxidation of the side chains of amino acid residue and peptide scission are principal products of PT-OX [17]. TCC accounts for PT-OX [26], thus confirming that IB-DBD-HVCP induced PT-OX in MPI. The RESPE, particularly ROS such as ozone (O_3_), generated by IB-DBD-HVCP, might have accelerated PT-OX in MPI. Reactive species can induce oxidative stress on lipids, nucleic acids as well as proteins [16]. The increased TCC in MPI with augmenting TT was attributed to a higher RESPE produced. In the present study, O_3_ was found to increase with increasing TT (data not shown). Zhang et al. [33] documented the increased TCC for bighead carp fillets subjected to ozone treatment in an ozone concentration manner. An increasing number of carbonyl groups can occur via the production of free radicals, mediated by the abstraction of hydrogen induced by the attack of RESPE on protein methylene carbon [34]. Estévez [17] documented the increased formation of carbonyl groups mediated by the oxidation of side chains of proline, arginine, threonine, and lysine. 

Oxidation of glutamyl residues related to fragmentation or peptide cleavage and α-amidation could also produce protein-bound carbonyl [17,26]. Similarly, the binding of non-protein carbonyl compounds from lipid oxidation products including malondialdehyde and 4-hydroxy-2-nonenal via Michael addition to the side chain of amino acid including lysine, cysteine sulfhydryl, and histidine imidazole amino groups could occur [35]. Additionally, Delles and Xiong [36] reported that the binding of malondialdehyde to myosin could induce the production of protein–protein cross-linked polymers. These changes might induce PT-OX of MPI. An increased TCC was documented for mackerel fillets treated with IB-DBD-HVCP generated by air at 80 kV for 5 min when compared with the CON [37]. These results suggested that IB-DBD-HVCP effectively induced PT-OX in MPI.

Surface hydrophobicity (SHP) of MPI without and with IB-DBD-HVCP treatment for various TT was different, in which the values of 947.84–1484.6 were attained (*p* < 0.05). Initially, the SHP of MPI increased when subjected to IB-DBD-HVCP treatment for 5 min (DBD-HVCP5). Subsequently, SHP began to decrease with increasing TT (*p* < 0.05). The alterations in SHP confirmed that IB-DBD-HVCP induced structural changes in MPI. Generally, the hydrophobic amino acid residues are situated internally in the protein molecules [38] and the augmentation in SHP is related to the exposed hydrophobic groups of protein molecules [33]. Therefore, the RESPE produced by IB-DBD-HVCP could attach to the protein molecules and more likely induced structural modification to some extent. Attri et al. [39] documented that structural changes occurred after proteins were treated with plasma generated with different working gases including Ar, air, and N_2_ at 60 Hz and 60 V. SHP increased in bighead carp protein subjected to ozone treatment in an ozone concentration manner [33]. The decrease in SHP for MPI subjected to IB-DBD-HVCP treatment for more than 5 min might be associated with the aggregation of proteins, thus shielding its hydrophobic domains to react with ANS. Therefore, IB-DBD-HVCP induced the denaturation of protein to some extent, but aggregation also occurred, particularly when exposed to IB-DBD-HVCP for a longer TT. 

There was a difference in the total sulfuryl content (TSHC) of MPI when subjected to IB-DBD-HVCP treatment for different TT (*p* < 0.05). Generally, the TSHC of MPI was found to decrease with increasing TT. This might be related to the enhanced oxidation of free sulfhydryl groups in fish muscle proteins, which are easily oxidized to disulfide bonds, hence resulting in a lower TSHC [24,40]. Sulfur containing amino acids, particularly cysteine, are prone to oxidation [41] and these modifications can alter the physico-chemical and functional properties of protein [16]. Sulfhydryl groups are oxidized by ROS and converted to disulfide bonds and other thiol oxidation products including sulfonic sulfinic, and sulfenic as well as thiosulfinates [42]. The formation of intermolecular disulfide bonds can be mediated by the oxidation of cysteine molecules between neighboring protein chains, depending on the extent of oxidation [43]. The decrease in TSHC was in line with the increase in TCC. Additionally, the induced protein aggregation might be associated with the masking of the sulfhydryl groups, which makes it unavailable for determination [40]. Furthermore, the changes in the protein conformation induced by IB-DBD-HVCP might have exposed the sulfhydryl groups, thus increasing the disulfide bond through oxidation [24]. Therefore, the treatment with IB-DBD-HVCP had a profound impact on the sulfhydryl content of MPI.

#### 3.1.2. Fourier Transform Infrared (FTIR) Spectra

**Figure 1:**
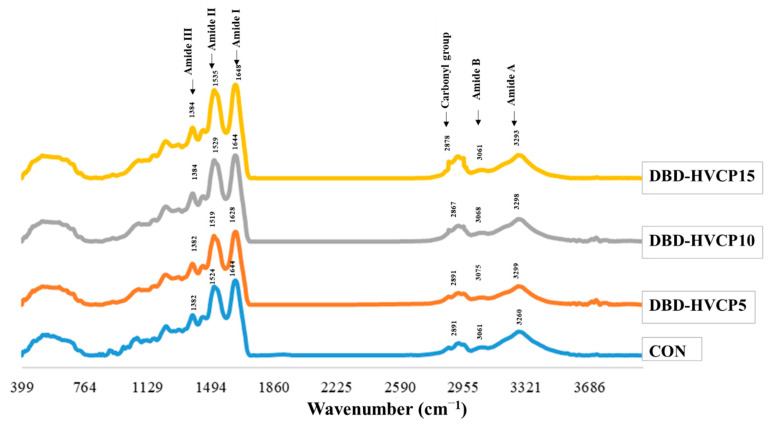
Fourier transform infrared (FTIR) spectra of Asian sea bass myofibrillar protein isolate without and with in-bag dielectric barrier discharge high voltage cold plasma (IB-DBD-HVCP) for different treatment times. CON: Asian sea bass myofibrillar protein isolate without DBD-HVCP treatment, DBD-HVCP5, DBD-HVCP10, and DBD-HVCP15: Asian sea bass myofibrillar protein isolate with DBD-HVCP for 5, 10, and 15 min, respectively. Gas mixture of argon/oxygen (90:10) was used as the working gas for DBD-HVCP.

FTIR spectra of MPI without and with IB-DBD-HVCP treatment for different TT are depicted in Figure 1. The oxidation status as well as the functional groups of the samples were identified using FTIR spectra. Irrespective of IB-DBD-HVCP treatment, the spectra of all the samples were similar in pattern, however slight differences in wavenumber and amplitudes could be observed as a function of TT. The amide I band was the major peak and was observed at 1644, 1628, 1644, and 1648 cm^−1^ for CON, DBD-HVCP5, DBD-HVCP10, and DBD-HVCP15, respectively, which is primarily the C=O stretching motion [44]. It was noted that wavenumber of DBD-HVCP5 was shifted to the lower wavenumber, indicating that there was interaction between C=O with adjacent functional groups. The amide I band components are widely used as probes for the conformation of proteins [45]. Amide II, which is derived mainly from in-plane NH bending and from the CN stretching vibration, occurred at 1524, 1519, 1529, and 1535 cm^−1^ for CON, DBD-HVCP5, DBD-HVCP10, and DBD-HVCP15, respectively. The shift of wavenumbers of samples treated with IB-DBD-HVCP for different TT suggested that the functional groups underwent interaction or became free form, depending on TT. However, the slightly higher amplitude of these bands, particularly the amide I band in DBD-HVCP10 and DBD-HVCP15 might indicate conformational changes, mainly due to fragmentation, thus liberating the functional groups of proteins. Based on the result, the change in wavenumber of amide I indicated the alteration of MPI as affected by IB-DBD-HVCP TT.

Additionally, amide A (3260, 3299, 3298, and 3293 cm^−1^), amide III (1384, 1384,1381, and 1382 cm^−1^), and amide IV (620, 622, 609, and 625 cm^−1^) were observed for CON, DBD-HVCP5, DBD-HVCP10, and DBD-HVCP15, respectively [46]. Increase in wavenumber of amide A, representing NH_2_, could suggest the release of free amino groups caused by the fragmentation or degradation of proteins as induced by active species [47]. The presence of aldehydes was confirmed by the C–H stretching frequency in FTIR. This characteristic C–H frequency combined with carbonyl oxygen produces its stretching vibration at 2900–2700 cm^−1^ [27]. The higher amplitude found in the IB-DBD-HVCP treated samples at this wavenumber confirmed the occurrence of protein oxidation, which was mediated by the RESPE generated by IB-DBD-HVCP. These findings correlated with the increasing TCC (Table 1). Overall, the FTIR results revealed that IB-DBD-HVCP had an influence on the functional groups of myofibrillar proteins in MPI, thus, some conformational changes were observed, especially when a longer TT of IB-DBD-HVCP was used.

### 3.2. Effect of IB-DBD-HVCP Treatment on the Gel Properties of MPI

#### 3.2.1. Breaking Force (BF) and Deformation (DF) 

**Figure 2:**
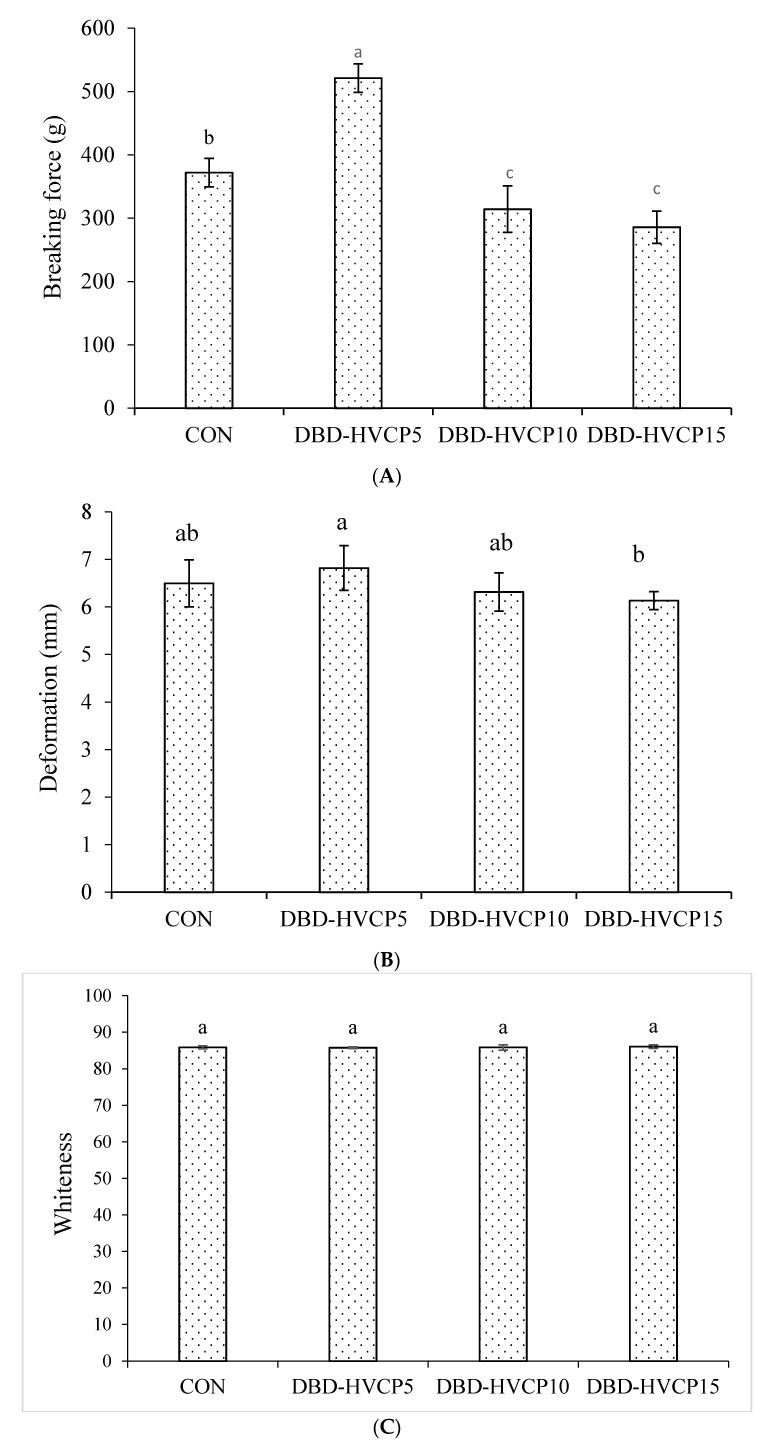

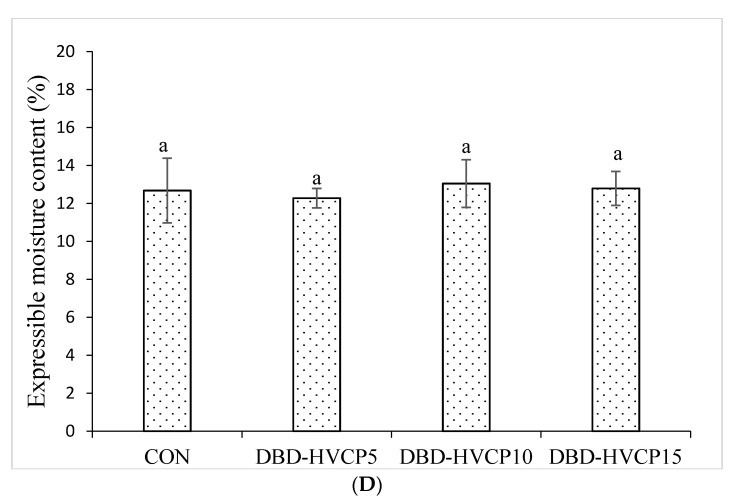
Breaking force (**A**), deformation (**B**), whiteness (**C**), and expressible moisture content (**D**) of gels from Asian sea bass myofibrillar protein isolate without and with IBDBD-HVCP for different treatment times. Bars represent the standard deviation (*n* = 3). Different lowercase letters on the bars indicate significant difference (*p* < 0.05). Caption: see Figure 1.

BF and DF of gels prepared from MPI without and with IB-DBD-HVCP treatment for various TT are presented in Figure 2A,B, respectively. BF and DF were highest for gels prepared from DBD-HVCP5 and were lowest for gels prepared from DBD-HVCP10 and DBD-HVCP15. This result was in line with the report of Zhang, Xue, Li, Wang, Yang and Xue [33], who documented increases in BF and DF of gels prepared from myofibrillar proteins recovered from bighead carp treated with O_3_ at 5.1 ppm when compared to the CON. A subsequent decrease in BF and DF was also found for samples treated with a high concentration of O_3_ (7.6 ppm) [33]. This indicated that IB-DBD-HVCAP, particularly for a short TT could improve the gel properties of MPI, whereas poor gel properties were evident at a longer TT. The gel forming property of myofibrillar proteins is influenced by hydrophobic and hydrophobic interactions, solubilization during grinding as well as reactive sulfhydryl content [43]. The increased BF and DF in DBD-HVCP5 might be associated with increased surface hydrophobicity (Table 1). As a result, enhanced hydrophobic–hydrophobic interaction can be attained. Moreover, gel formation could be enhanced by the covalent bonding between carbonyl groups in DBD-HVCP5 with amino groups during gel formation, thereby enhancing protein interaction [33]. Conversely, the lower BF and DF in MPI treated with DBD-HVCAP for 10 and 15 min might be associated with the reduced surface hydrophobicity as well as lower total sulfhydryl groups associated with aggregation. This could lower the solubility of proteins and affect the gel property of the MPI [48]. The lower BF and DF in the aforementioned samples might also be related with high PT-OX, which might induce fragmentation, in which short chain peptides could not form strong gels. Therefore, the properties of the surimi gel including BF and DF were influenced by IB-DBD-HVCP treatment. 

#### 3.2.2. Whiteness and Expressible Moisture Content (EMC)

Whiteness (Figure 2C) and expressible moisture content (EMC, Figure 2D) of gels prepared from MPI without and with IB-DBD-HVCP treatment for different TT were not significant, in which the values of 85.77–86.10 and 12.28–13.05, respectively, were obtained. The water holding capacity (WHC) of the gel is inversely associated with its EMC. Generally, WHC is directly related with the BF of the gels. Although BF was highest in DBD-HVCP5, the EMC of the gel was not different from the CON and remaining treated samples (*p* > 0.05). This was likely due to the formation of a disordered gel network by the denatured or aggregated proteins, which could hold more water [49]. Generally, all the gel showed high whiteness, regardless of IB-DBD-HVCP treatment, which is more likely due to the removal of all the pigments, especially myoglobin during the washing and protein isolate preparation. Moreover, all the samples showed similar EMC, indicating a high amount of water imbibed in the network, which can lead to the maximum scattering of light. This can cause a higher whiteness of all the gel samples. Therefore, it can be postulated that the IB-DBD-HVCP conditions used in this present study did not have a marked influence on the whiteness and WHC of the MPI gel.

#### 3.2.3. Protein Patterns 

**Figure 3:**
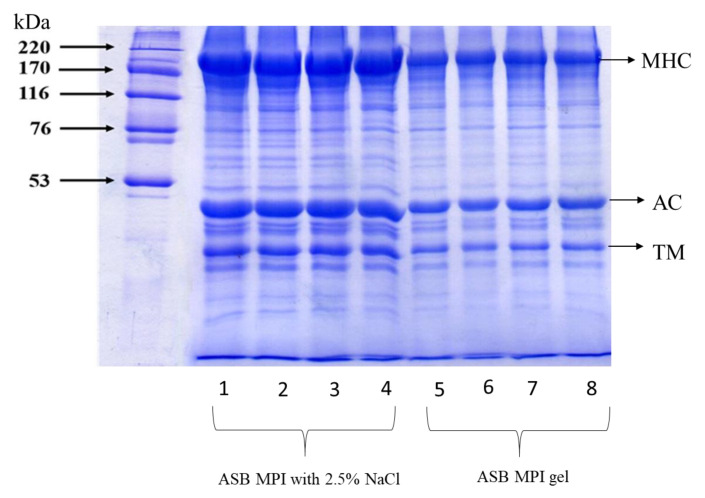
Protein patterns of gels from Asian sea bass myofibrillar protein isolate without and with IBDBD-HVCP for different treatment times. MHC: myosin heavy chain, AC: actin and TM: tropomyosin. Line 1 and 5: Con, Lane 2 and 6: DBD-HVCP5, Lane 3 and 7: DBD-HVCP10, Lane 4 and 8: DBD-HVCP15. Caption: see Figure 1.

Protein patterns of the MPI paste (containing 2.5% NaCl) and gel without and with IB-DBD-HVCP treatment at various TT are depicted in Figure 3. Generally, myosin heavy chain (MHC) and actin were the dominant proteins in MPI. These findings were in accordance with the report of Olatunde et al. [50]. After treatment with IB-DBD-HVCP for 5 and 10 min, the band intensity (BI) of MHC and actin of MPI were not different compared to that of the CON. A similar observation was recorded for bighead carp MPI treated with ozonated water at different concentrations [33]. However, when TT was increased to 15 min, there was a slight decrease in the BI of MHC and actin. The band intensity of MHC and actin of the aforementioned sample was decreased by 15.45 and 8.91%, respectively, when compared to that of the corresponding control. Olatunde, Benjakul, and Vongkamjan [50] and Olatunde, Benjakul, and Vongkamjan [32] documented a decrease in the BI of MHC and actin when ASB slices were subjected to IB-DBD-HVCP for a longer TT. The RESPE generated during IB-DBD-HVCP, particularly at a longer TT, might induce the fragmentation or degradation of proteins. The decreases in MHC and actin BI in porcine muscle myofibrillar protein were reported when treated with hydroxyl radicals [51]. Park et al. [52] found that MHC and actin had less resistance to oxidation because they are targets for oxidative reaction. Additionally, Stagsted et al. [53] documented that MHC and actin were more prone to oxidative damages, which can induce the polymerization or formation of aggregates [54]. This confirmed the degradation and fragmentation of proteins in MPI paste when subjected to IB-DBD-HVCAP, especially for a longer time.

Regardless of the IB-DBD-HVCP treatment, a reduction in the BI of MHC in gel, relative to that found in paste, was observed. This indicated that the setting phenomenon had taken place in the MPI paste. MHC is preferable for the setting phenomenon, in which a non-disulfide covalent bond is developed. A slight decrease was observed for actin. The result confirmed that MHC was a favorable substrate for the setting [55]. The result indicated that IB-DBD-HVCP had no profound impact on the protein pattern of gel when analyzed using SDS-PAGE under a reducing condition. 

#### 3.2.4. Dynamic Rheological Properties

**Figure 4:**
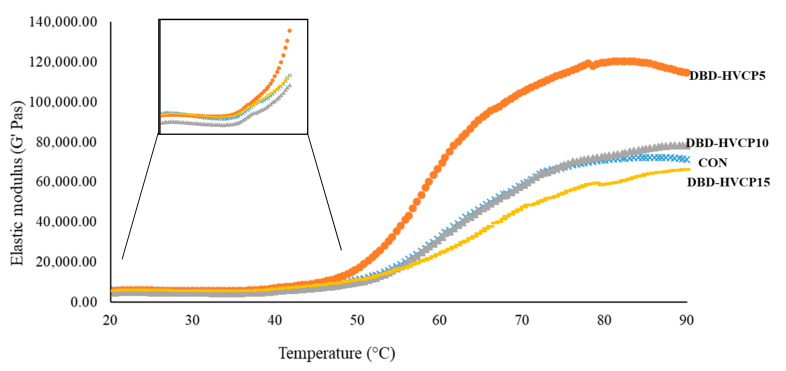
Elastic modulus of gels from Asian sea bass myofibrillar protein isolate without and with IBDBD-HVCP for different treatment times. Caption: see Figure 1.

The elastic modulus (G′) of MPI without and with IB-BDB-HVCP treatment for different TT during conversion from sol to gel as a function of temperature is depicted in Figure 4. During the formation of viscoelastic materials, the stored energy is referred to as G′ [29,31]. G′ remained constant for all the samples up to 50 °C. Subsequently, G′ began to gradually increase. Buamard and Benjakul [56] and Singh, Prabowo, Benjakul, Pranoto, and Chantakun [31] documented a decreased G′ at 50 °C for surimi paste prepared form sardine and spotted golden goatfish fish, which was associated with the endogenous proteases at this range of temperature. Nevertheless, this phenomenon was not apparent in this study. This might be related to the inactivation of protease during the alkali solubilization of MPI [57]. At this temperature, DBD-HVCP5 had the highest G′ when compared to the others. This suggested that the proteins exposed to IB-DBD-HVCP for 5 min more likely underwent entanglement with subsequent aggregation. Additionally, conformation changes induced by IB-DBD-HVCP for 5 min could favor aggregation via several bondings. The lower G′ reported for other samples might be related to the fragmentation or excessive aggregation of proteins, thus reducing the protein interaction [58]. Aggregated proteins more likely had poor solubility in salt used for grinding MPI. As a result, the fine and ordered gel network could not be formed during heat-induced gelation process. Additionally, oxidation induced by IB-DBD-HVCP, particularly when a longer time was used, might induce the cleavage of the proteins, thus leading to the formation of short chain polypeptides, which could not undergo interaction or aggregation effectively ascertained by poor gel formation [59]. When MPI was further heated, a continuous increase in G′ for all the samples was recorded up to 80 °C. The DBD-HVCP5 sample showed the highest G′, while DBD-HVCP15 showed the lowest G′ value. The result corresponded with the lowest BF (Figure 2A). The cross-linking of dissociated proteins was more likely responsible for the increment in G′ value. Moreover, the formation of a thermo-irreversible protein network could be induced by the denaturation of MHC [60]. The enhanced interaction of reactive groups or domains such as hydrophobic–hydrophobic interaction as well as the formation of disulfide bonds as a result of the unfolding of proteins also contributed to the increased G′ [49]. There was a gradual reduction in G′ value for the CON and DBD-HVCP5 samples when the temperature was above 80 °C. This was likely due to the breakdown of hydrogen bonding at high temperature [31,56,58]. Therefore, IB-DBD-HVCP treatment, particularly for 5 min, enhanced the proteins cross-linking in MPI, which resulted in gel formation with improved elasticity and strength.

#### 3.2.5. Microstructure 

**Figure 5:**
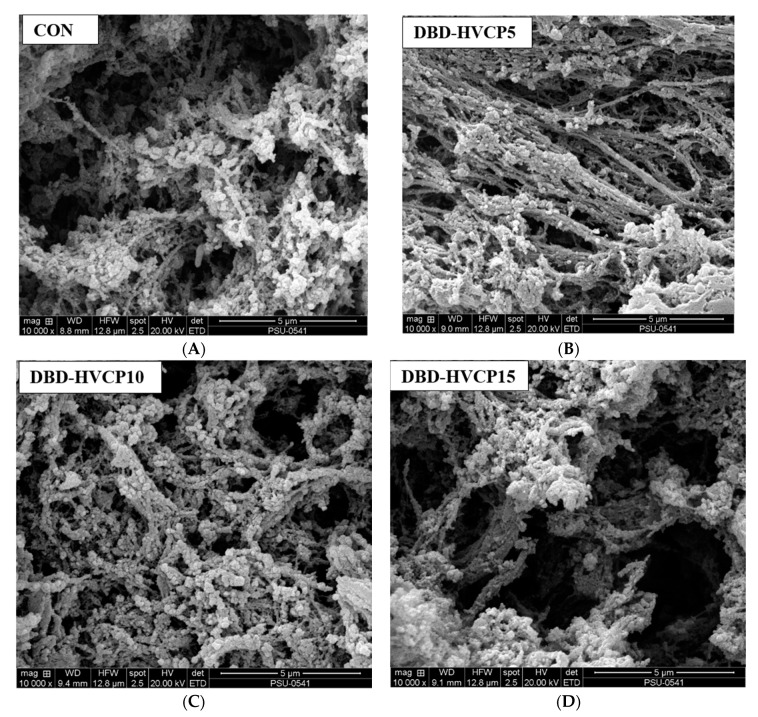
Scanning electron microscopic image of gels from Asian sea bass myofibrillar protein isolate without and with IB-DBD-HVCP for different treatment times. Magnification: 10,000×. Caption: see Figure 1. (**A**) CON; (**B**) DBD-HVCP5; (**C**) DBD-HVCP10; (**D**) DBD-HVCP15.

Microstructures of MPI without and with IB-DBD-HVCP treatment for different times are presented in Figure 5A–D. A coarser network with larger voids or cavities was found for CON. When MPI was subjected to IB-DBD-HVCP for 5 min, protein strands of the resulting gel had higher density in comparison with those found in the CON gel. Moreover, fine protein strands with higher connectivity were also noticed in DBD-HVCP5. This confirmed the effective cross-linking of myofibrillar strands via the modification of the protein structure mediated by IB-DBD-HVCP treatment for 5 min [61]. This corresponded with the high BF and G′ in DBD-HVCP5. Ngo et al. [62] documented high BF for gels with a denser gel network. Buamard and Benjakul [56] related higher interconnectivity of strands as well as density to high G′ and BF. When IB-DBD-HVCP TT was increased (greater than 5 min), the resulting MPI gel appeared to be coarser with larger voids when compared to the CON (Figure 5C,D), which signified the coagulation or lack of continuous connection between protein molecules. This was in accordance with the lower G′ and BF recorded for these samples. Therefore, the pre-treatment of MPI with IB-DBD-HVCP, particularly for 5 min, more likely induced the higher rigidity and connectivity of protein strands within the MPI gel networks.

## 4. Conclusions

IB-DBD-HVCP can induce protein oxidation in MPI as ascertained by slightly decreased sulfhydryl group content, augmented carbonyl content, and altered surface hydrophobicity, thus modifying the gel properties of the protein. However, these modifications of MPI induced by IB-DBD-HVCP when a TT of 5 min was used enhanced the crosslinking of protein with coincidental improved gel network compared to the control. When TT was further increased, the gelling properties of MPI were negatively affected. Nevertheless, the whiteness and EMC of the resulting gel were not influenced by the DBD-HVCP treatment, regardless of TT. Thus, IB-DBD-HVCP, particularly for 5 min, had a positive effect on the gelling properties of myofibrillar proteins in MPI from Asian sea bass. IB-DBD-HVCP could therefore be explored as a non-thermal processing technology for the protein improvement/modification. From the food authority regulations and market expectation standpoints, this technology can meet the demand for improving the techno-functional properties. However, this technology can only be fully exploited in foods when the process parameters such as the TT, working gas composition, applied voltage/power, and post-treatment time are optimized.

## Figures and Tables

**Table 1:** Total carbonyl content, surface hydrophobicity, and total sulfhydryl group content of Asian sea bass myofibrillar protein isolate without and with in-bag dielectric barrier discharge high voltage cold plasma (IB-DBD-HVCP) for different treatment times.

Samples	Total Carbonyl Content (nmol/g Protein)	Surface Hydrophobicity	Total Sulfhydryl Content (µmol/g Protein)
CON	1.16 ± 0.01 ^d^	1165.4 ± 2.21 ^c^	7.94 ± 0.20 ^a^
DBD-HVCP5	2.39 ± 0.07 ^c^	1484.6 ± 0.97 ^a^	6.53 ± 0.10 ^b^
DBD-HVCP10	4.76 ± 0.02 ^b^	1380 ± 1.04 ^b^	5.28 ± 0.02 ^c^
DBD-HVCP15	7.93 ± 0.01 ^a^	947.84 ± 1.41 ^d^	2.94 ± 0.05 ^d^

Values represent mean and standard deviation (*n* = 3). Different superscripts within the same column indicate significant differences (*p* ˂ 0.05). CON: Asian sea bass myofibrillar protein isolate without DBD-HVCP treatment, DBD-HVCP5, DBD-HVCP10, and DBD-HVCP15: Asian sea bass myofibrillar protein isolate with DBD-HVCP for 5, 10, and 15 min, respectively. Gas mixture of argon/oxygen (90:10) was used as working gas for DBD-HVCP.

## Data Availability

Data sharing not applicable.
